# Immune and Autoimmune Mechanisms in Autism Spectrum Disorder: Linking Molecular Pathways to Neuropsychological Trajectories Across the Lifespan

**DOI:** 10.3390/ijms27188407

**Published:** 2026-09-21

**Authors:** Rosa Gemma Viterbo, Rosalba Tritto, Dario Sardella, Pietro Iaffaldano, Andrea De Giacomo

**Affiliations:** 1 Child Neuropsychiatry Unit, Department of Precision and Regenerative Medicine, Jonic Area University of Bari “Aldo Moro”, 70124 Bari, Italy; rossellaviterbo4@gmail.com (R.G.V.); andrea.degiacomo@uniba.it (A.D.G.); 2 Department of Translational Biomedicine and Neurosciences-DiBraiN, University of Bari “Aldo Moro”, 70124 Bari, Italy

**Keywords:** autism spectrum disorder, neuroimmunology, autoimmunity, maternal immune activation, maternal autoantibodies, cytokines, microglia, neuroinflammation, neuropsychology, lifespan development

## Abstract

Autism spectrum disorder (ASD) is a heterogeneous neurodevelopmental condition with wide differences in cognitive, behavioral, and adaptive outcomes across the lifespan. Current evidence suggests that part of this heterogeneity may be related to interactions among genetic susceptibility, environmental exposures, and immune processes acting during important periods of brain development. This narrative review examines the molecular and cellular mechanisms through which immune dysregulation and autoimmunity may contribute to neurodevelopmental variability in ASD. It also explores possible links with age-related neuropsychological trajectories. Main topics include maternal immune activation, maternal brain-reactive autoantibodies, cytokine signaling, peripheral immune cell changes, microglial and astrocytic function, complement-mediated synaptic remodeling, and gut–immune–brain pathways. These mechanisms are discussed in relation to language, attention, executive functions, sensory processing, social cognition, emotional regulation, adaptive functioning, and psychiatric comorbidity from childhood to adulthood. Current evidence supports immune dysfunction as a possible modifier of ASD phenotypes rather than a universal or isolated cause. Direct longitudinal evidence linking specific immune profiles to defined neuropsychological trajectories is still limited, especially in adults. Integrating molecular, immunological, and neuropsychological data may help identify biologically meaningful ASD subgroups and support future biomarker development, developmental stratification, and more personalized therapeutic approaches.

## 1. Introduction

Autism spectrum disorder (ASD) is a lifelong neurodevelopmental condition characterized by differences in social communication and interaction, together with restricted or repetitive patterns of behavior, interests, or activities. However, people with ASD show very different developmental and clinical profiles. Cognitive ability, language, adaptive functioning, sensory processing, psychiatric comorbidity, and long-term outcomes may vary greatly between individuals [1,2].

ASD also has a complex biological basis. Genetic studies show an important role for both rare variants and common polygenic risk. Many ASD-related genes are involved in synaptic function, transcription, chromatin regulation, and early brain development. At the same time, genetic factors do not explain all clinical variability. Environmental and biological exposures during prenatal and early postnatal development may interact with genetic susceptibility and change developmental pathways [3,4].

The immune system is one possible biological modifier. Immune molecules and immune cells have normal roles in brain development. Cytokines, chemokines, complement proteins, microglia, astrocytes, and peripheral immune signals contribute to neuronal maturation, synapse formation, synaptic remodeling, and neural circuit organization. For this reason, changes in immune signaling during sensitive developmental periods may influence brain development even when they do not produce a classic inflammatory disease [5,6].

Several lines of evidence suggest immune differences in at least a subgroup of autistic individuals. These include altered peripheral cytokine profiles, changes in immune cell populations, higher rates of some immune-mediated conditions, and postmortem signals of glial and inflammatory changes. Prenatal mechanisms are also important. Maternal immune activation and maternal IgG antibodies that react with fetal brain proteins provide possible pathways through which maternal immune status may influence fetal neurodevelopment [7,8,9].

It is important to use clear definitions. Immune dysregulation is a broad term that includes altered immune cell function or signaling. Systemic inflammation refers to inflammatory activity outside the central nervous system. Neuroinflammation refers to inflammatory processes within the brain or spinal cord. Maternal immune activation describes the maternal inflammatory response during pregnancy. Autoimmunity refers to immune responses directed against self-antigens. These concepts overlap but are not identical, and an association with ASD does not by itself prove causality.

A lifespan approach is also needed. Neuropsychological profiles in ASD are not fixed. Language, sensory processing, attention, executive control, social cognition, emotional regulation, and adaptive functioning can change with age and environmental demands. The biological factors that are relevant in early childhood may not be the same as those that influence adolescence or adulthood.

Most reviews examine neuroimmunology and neuropsychology separately. As a result, it is often unclear whether immune findings are directly related to specific cognitive or adaptive outcomes. The present review therefore examines immune and autoimmune mechanisms in ASD and asks how strongly they can be linked to neuropsychological variability across development. Human clinical evidence, postmortem findings, animal models, and cellular studies are considered separately to avoid overinterpretation.

## 2. Methods of the Narrative Review

### 2.1. Review Design and Scope

This article was designed as a narrative review with a structured literature search. The aim was not to perform a meta-analysis, but to identify and integrate human and experimental evidence on immune mechanisms, autoimmunity, and neuropsychological development in autism spectrum disorder (ASD). The overall approach was informed by the SANRA recommendations for narrative reviews [10].

### 2.2. Search Strategy and Screening Workflow

The literature search was performed in PubMed and was organized into complementary thematic modules rather than one single broad query. All PubMed searches used the filter “last 5 years”. The modules addressed maternal immune activation, maternal brain-reactive autoantibodies, peripheral cytokines and immune cell profiles, microglial and neuroinflammatory mechanisms, complement-mediated synaptic remodeling, autoimmune comorbidity, direct immune–neuropsychological associations, and adult or aging-related evidence. The original PubMed History log was not retained; therefore, Supplementary Table S1 reports reconstructed search strings corresponding to the documented search modules and saved screening files.

The five-year filter was used for the structured searches. Older landmark papers were retained only when they were already part of the original manuscript or were identified by citation chasing to support historical or validation-specific claims.

The initial broad PubMed retrieval contained 561 records. Title screening retained 127 records for abstract assessment: 69 were classified as core evidence and 58 as supporting mechanistic evidence. A further 24 records were retained only for background or citation chasing, while 410 were excluded at title level. At abstract level, 47 papers were prioritized for routine full-text retrieval, 27 were retained as selective support, and 53 were considered background or no-full-text-priority sources.

Additional targeted searches were used to address gaps identified during drafting. Three focused searches on direct immune–neuropsychological links, maternal autoantibody phenotypes, and developmental timing yielded 29 unique records after removal of one duplicate; 12 were prioritized for full-text review, 8 were retained for selective use, and 9 were excluded. A separate adult/aging search retrieved 36 records, of which five were selected for detailed full-text analysis. These targeted searches overlapped with the initial search and their counts should therefore not be summed as independent study totals. A structured evidence matrix was finally constructed for 32 full-text papers selected for detailed mechanistic or lifespan synthesis.

Screening was used as a structured aid for a narrative review and was not performed as a formal dual-independent systematic review process. No inter-rater reliability statistic was calculated. A PRISMA-style diagram is provided as Supplementary Figure S1 only to improve transparency of the search and selection process.

### 2.3. Eligibility Criteria and Data Extraction

Eligible publications included systematic reviews and meta-analyses, cohort studies, case–control studies, cross-sectional biomarker studies, postmortem or transcriptomic studies, molecular imaging studies, and relevant animal or cellular studies. Studies were selected when they addressed physiological immune regulation, prenatal immune exposure, maternal antibodies, peripheral innate or adaptive immunity, cytokines or chemokines, microglia, astrocytes, complement, neuroinflammation, autoimmunity, or direct links with cognitive, behavioral, adaptive, or psychiatric outcomes.

Studies were excluded when they were not relevant to ASD, when immune findings had no clear neurodevelopmental context, when they were isolated case reports without major mechanistic value, or when they focused only on treatment efficacy without relevant biological information. Reviews were mainly used to map the field and identify key primary studies. For the full-text evidence set, information was extracted on study design, population or model, age, ASD characterization, biospecimen or tissue, immune pathway or biomarker, neuropsychological or behavioral outcomes, main results, covariates, limitations, and translational relevance.

### 2.4. Operational Evidence Categories

Evidence was classified into four operational categories. Direct clinical evidence required an immune exposure, biomarker, or immune cell measure and a neuropsychological or developmental outcome to be assessed within the same human cohort, including prospective prenatal or neonatal exposure studies with later phenotyping. Indirect clinical evidence included human studies relevant to ASD biology or neuropsychology in which the immune and neuropsychological components were not directly linked in the same analysis. Experimental evidence included animal, cellular, organoid, or other model systems in which an immune pathway was manipulated or tested mechanistically. Hypothesis-generating evidence included exploratory multi-omics, postmortem, imaging, or integrative findings that suggested a pathway but did not establish a direct clinical or experimental causal link. These categories describe directness of evidence and are not a formal grading system.

### 2.5. Methodological Appraisal and Weighting of Evidence

Because this review included several different study designs, one risk-of-bias tool could not be applied appropriately to all papers. We therefore used a design-adapted structured appraisal. For human observational biomarker studies, the main domains were participant selection, biomarker measurement, outcome measurement, control of confounding, missing data, multiple testing, and temporal direction. For systematic reviews and meta-analyses, we considered search completeness, study-level risk of bias, heterogeneity, and sensitivity analyses. For experimental studies, we considered model relevance, sample size, randomization or blinding when reported, outcome reporting, reproducibility across models, and the translational distance from human ASD. These domains were used to assign an overall narrative confidence level (higher, moderate, or lower) to the key findings in Supplementary Table S2. These labels are not GRADE ratings.

We chose this structured narrative appraisal because the 32 full-text papers included highly heterogeneous designs, including meta-analyses, prospective and cross-sectional human studies, postmortem and transcriptomic analyses, imaging studies, animal models, and cellular systems. A single formal risk-of-bias instrument, including ROBINS-I, would not be valid across all these designs and could create a false impression of comparability. Applying several different formal tools would also produce scores that are not directly comparable across evidence types. For this reason, we used design-specific appraisal domains and an overall narrative confidence judgment, while keeping human association, experimental causality, and translational distance separate. This approach was intended to improve transparency without converting the review into a formal systematic review framework.

Greater weight was given to prospective human studies, systematic reviews or meta-analyses with transparent methods, larger and well-characterized cohorts, standardized neuropsychological measures, appropriate control of confounding and multiple testing, and replicated or convergent findings. Particular attention was given to age, sex, intellectual disability, language level, epilepsy, gastrointestinal or allergic conditions, sleep, medication, body mass index, recent infection, biospecimen type, assay platform, sample handling, and timing of exposure or measurement. Human association and experimental causality were kept separate throughout the synthesis.

When meta-analyses or reviews overlapped substantially, we prioritized the more recent and methodologically transparent synthesis, while using older or overlapping reports only when they added a distinct population, outcome, or mechanistic perspective. We did not count overlapping syntheses as independent evidence. When results were conflicting, we compared differences in sample composition, age, biospecimen, assay platform, statistical methods, and study quality rather than selecting one result as definitive. No additional quantitative pooling was performed.

Practical recommendations for the design and reporting of immune-biomarker studies in ASD are summarized in Table 1.

## 3. ASD Heterogeneity and the Rationale for a Neuroimmune Framework

### 3.1. From a Behavioral Diagnosis to Biological Heterogeneity

ASD is defined by behavior, but the same diagnosis can include very different developmental profiles. People with ASD may differ in intellectual ability, language, adaptive functioning, sensory processing, psychiatric comorbidity, and long-term outcome. This clinical variability is consistent with biological heterogeneity rather than one single disease mechanism.

Neuroimmune mechanisms may contribute to this heterogeneity. Peripheral biomarker studies, postmortem studies, transcriptomics, epidemiological cohorts, maternal immune exposure studies, and experimental models all show that immune-related changes can occur in ASD. However, the evidence does not support one immune phenotype shared by all autistic individuals. Immune findings vary with age, sex, tissue, genetic background, environmental context, and comorbidities [12,13].

For this reason, ASD should not be described as a general inflammatory or autoimmune disorder. Immune processes are better viewed as possible developmental modifiers. Their effects may depend on timing, intensity, biological susceptibility, and interaction with other molecular pathways.

### 3.2. Peripheral Immune Signatures

Recent meta-analyses provide strong evidence for group-level differences in peripheral inflammatory markers. Volk et al. [14] reviewed 98 studies and reported higher levels of several cytokines in autistic groups, including IL-1β, IL-4, IL-6, IL-8, IFN-γ, TNF-α, CXCL1/GRO-α, and macrophage migration inhibitory factor. Chen et al. [15] also found group-level differences in CRP, IL-6, IL-8, IL-1β, and BDNF.

These findings are important, but they do not define a diagnostic biomarker. There is large overlap between groups, and results are influenced by age, sex, assay method, biospecimen, medication, body mass index, sleep, infection, and comorbidity. Volk et al. also found no strong evidence that cytokine levels consistently tracked autistic traits within autistic samples.

Cellular studies show the same pattern. Li et al. [16] reported increased classical and non-classical monocytes and increased monocyte IL-6 production in children with ASD. Classical monocyte levels were negatively associated with adaptive behavior, fine-motor, and personal–social developmental quotients. This is direct evidence of an immune–developmental association, but the cross-sectional design does not show causality.

### 3.3. Central Immune and Glial Heterogeneity

Postmortem and transcriptomic studies also show immune and glial changes in the central nervous system. A common pattern is relative upregulation of immune or glial programs together with downregulation of neuronal and synaptic programs. However, these findings vary by brain region, developmental stage, cellular composition, and method.

Microglia are a clear example. Older studies often used the broad term ‘microglial activation’. Recent evidence suggests a more complex picture. Microglial states differ by region, age, sex, metabolic context, and disease model. There is no single activated microglial state that defines ASD [12]. Astrocytes also contribute to synaptic, metabolic, ionic, and inflammatory regulation, but causal evidence is still mainly experimental [17,18].

### 3.4. Immune-Related Subgroups

Recent models propose immune-related ASD subgroups based on inflammation, impaired immune regulation, autoimmunity, gut–immune changes, maternal immune activation, and other mechanisms. These models are useful for research, but they are not validated clinical categories.

A safer framework is to consider immune mechanisms as probabilistic modifiers of neurodevelopment. Different combinations of genetic risk, prenatal exposure, peripheral and central immune signaling, glial function, metabolism, and environmental factors may lead to similar behavioral diagnoses. This model supports the study of immune biology in relation to specific developmental outcomes rather than diagnosis alone.

**Figure 1:**
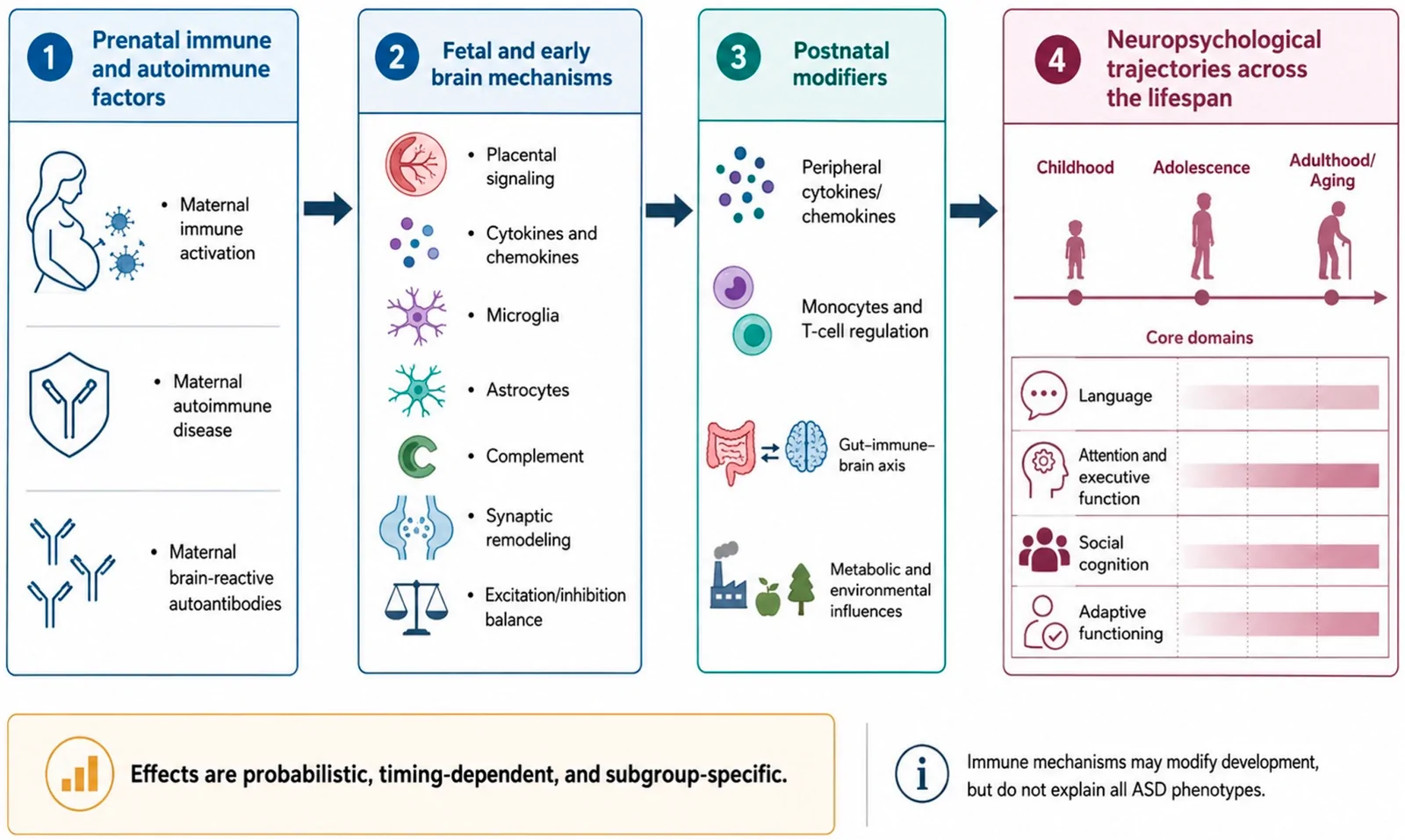
Developmental neuroimmune framework in autism spectrum disorder (ASD). Immune mechanisms may modify neurodevelopment through prenatal and postnatal pathways, with effects that depend on timing, biological context, and subgroup susceptibility.

As shown in Figure 1, color coding indicates developmental timing and conceptual level: blue represents prenatal and fetal/early-brain processes, teal represents postnatal modifiers, and burgundy represents neuropsychological trajectories across later development. The figure is conceptual and does not use color intensity as a quantitative evidence grade. Strength and directness of evidence for individual pathways are summarized separately in the evidence-synthesis tables below to avoid implying greater certainty than the data support.

### 3.5. Convergence Between ASD Risk Genes and Immune Cell Biology

A stronger neuroimmune model of ASD should include genetic risk. Many ASD-associated genes were first studied in neurons, but several also influence microglial or glial functions. This creates a possible point of convergence between genetic susceptibility and immune regulation. Human iPSC-derived microglia and organoid systems are especially useful because they can test cell-intrinsic effects of ASD-related variants in a human developmental context [19,20].

Recent studies provide examples of this convergence. A CRISPR-interference screen of ASD risk genes in human microglia identified ADNP as a regulator of endocytosis and synaptic pruning [20]. In another multi-species model, SCN2A deficiency altered microglial pruning through complement C3 and increased elimination of post-synaptic elements in both mice and human cerebral organoids [21]. These findings suggest that neuronal risk genes can change the neuroimmune environment and that microglia can become part of the downstream disease mechanism rather than only a secondary inflammatory response.

These findings are mechanistically informative, but they come from gene-specific experimental systems. They show that ASD-related variants can influence microglial and complement pathways, not that the same pathway is active in all autistic individuals or that it predicts a specific human neuropsychological phenotype.

MEF2C provides a second example. MEF2C is an ASD-related transcription factor with important functions in both neural development and microglial homeostasis. In human pluripotent stem cell-derived microglia, loss of MEF2C increased inflammatory responses and altered phagocytic, lysosomal, and lipid-handling functions [22,23]. These data support a model in which transcriptional risk and immune cell state can converge on shared pathways such as phagocytosis, lipid metabolism, NF-κB signaling, and synaptic remodeling. However, these mechanisms are based mainly on experimental systems and cannot yet be used to define a common immune mechanism across idiopathic ASD.

## 4. Physiological Roles of the Immune System in Brain Development

### 4.1. Neuroimmune Signaling in Normal Development

The immune and nervous systems are closely linked during development. Cytokines, chemokines, complement proteins, microglia, astrocytes, and peripheral immune signals take part in normal brain maturation. They help regulate cell proliferation, differentiation, neuronal migration, synapse formation and elimination, glial maturation, vascular function, and circuit refinement.

These effects are strongly time-dependent. The same immune signal may have different effects depending on concentration, cell type, developmental stage, and tissue context. Therefore, prenatal immune activation, neonatal immune markers, childhood inflammation, and adult immune changes should not be treated as the same biological event.

### 4.2. Microglia and Synaptic Refinement

Microglia are resident immune cells of the central nervous system. During development they interact with neurons, synapses, astrocytes, and extracellular signals. They take part in phagocytosis, trophic support, synapse formation and elimination, and activity-dependent circuit remodeling.

Complement signaling is one pathway that links immunity with synaptic refinement. Complement proteins can mark selected synaptic elements for recognition and engulfment by microglia. Other pathways, including phosphatidylserine signaling and inhibitory ‘do-not-engulf’ signals, also regulate this process.

In ASD-related models, both excessive and insufficient or mistargeted synaptic elimination have been described. It is therefore better to speak of dysregulated synaptic remodeling rather than generalized excessive pruning.

### 4.3. Astrocytes and Neuroimmune Regulation

Astrocytes support synapse formation, regulate extracellular glutamate and potassium, provide metabolic support, and contribute to blood–brain barrier homeostasis. They can also respond to inflammatory signals and influence neurons and microglia.

Recent reviews of human tissue, induced pluripotent stem cell systems, organoids, and animal models suggest that astrocytic abnormalities may affect synaptic development, excitatory–inhibitory balance, metabolism, and inflammatory signaling in ASD-related conditions [17]. These findings are biologically important, but much of the strongest causal evidence remains experimental.

### 4.4. Cytokines and Chemokines as Developmental Signals

Cytokines and chemokines are not only markers of inflammation. They can influence neuronal and glial development through receptors expressed by many cell types. Their effects depend on timing and biological context.

Kim et al. [24] found that neonatal chemokine profiles were related to later ASD or developmental-delay classification. Higher neonatal CCL23/MPIF-1 was also associated with better performance in several developmental domains among children later diagnosed with ASD. This result shows why higher immune signaling should not always be interpreted as a negative developmental factor.

### 4.5. From Physiological Function to Developmental Disturbance

The main issue is not the presence of immune activity, because immune activity is part of normal development. Problems may arise when timing, duration, intensity, cellular targeting, or regulatory balance are altered. Genetic susceptibility, fetal sex, placental biology, maternal immune state, metabolic context, and later environmental exposures may all change the effect of the same immune stimulus.

## 5. Prenatal Immune and Autoimmune Mechanisms

### 5.1. Maternal Immune Activation

Maternal immune activation (MIA) describes the maternal inflammatory response during pregnancy, for example during infection or inflammatory disease. Epidemiological and experimental work has made MIA one of the most studied prenatal neuroimmune mechanisms in ASD and other neurodevelopmental disorders.

Animal models show that maternal inflammatory signals can change placental signaling, fetal cytokine environments, microglial maturation, synaptic development, neurotransmitter systems, and later behavior. IL-6 and IL-17A pathways, including maternal Th17 responses, have received particular attention. These mechanisms are strong in experimental models but are not established causal pathways in human ASD [25,26].

MIA models also show important heterogeneity. The effect depends on immune stimulus, dose, gestational timing, maternal microbiota, placental response, and fetal sex. Schaer et al. [27] showed different juvenile susceptibility and resilience profiles after MIA, with sex-dependent adult trajectories. This supports a probabilistic model rather than a deterministic one.

It is also important to separate different MIA models. Poly(I:C) mainly models antiviral-like innate signaling, whereas LPS activates bacterial-like Toll-like receptor pathways. Cytokine profiles, placental responses, fetal brain effects, and behavioral outcomes can differ between these paradigms. The same is true for timing and dose. Therefore, IL-6 or IL-17A should not be presented as universal MIA pathways. They are strong mechanistic findings in selected experimental settings, while the corresponding causal chain in human pregnancy remains uncertain [25,26,27].

Across MIA paradigms, the most reproducible level of evidence is not one single cytokine or behavioral phenotype, but the broader sequence of maternal inflammatory activation, altered placental or fetal signaling, and later changes in neurodevelopmental outcomes. By contrast, the dominant cytokine pathway, affected brain region, sex effect, behavioral domain, and persistence of immune changes are more model-dependent. Poly(I:C) studies often emphasize antiviral-like signaling and IL-6/IL-17A-related pathways, whereas LPS models more strongly engage bacterial-like TLR4 signaling and may produce different cytokine and metabolic profiles. Longitudinal studies also show that persistent behavioral effects do not require persistent inflammatory markers [25,26,27,28,29]. This distinction between robust developmental principles and model-specific molecular effects is important when translating MIA findings to human ASD.

### 5.2. Maternal Autoimmune Disease

Human epidemiology also suggests that maternal immune state may be relevant. Zhu et al. [30] examined maternal rheumatoid arthritis and offspring ASD in two national birth cohorts and a meta-analysis. Maternal rheumatoid arthritis present before delivery was associated with a modest increase in ASD risk. Rheumatoid arthritis diagnosed after delivery was not similarly associated.

This temporal pattern supports the importance of the prenatal period but does not identify the mechanism. Cytokines, autoantibodies, shared genetics, disease activity, medication, or other correlated factors may contribute.

### 5.3. Maternal Inflammatory Proteins

Prospective measurement of maternal inflammatory proteins may provide more direct information than maternal diagnosis alone. Wang et al. [31] measured 92 inflammatory proteins during pregnancy and assessed offspring outcomes at age 10 years. A multivariate inflammatory pattern was associated with neurodevelopmental disorders, especially ASD. However, associations with executive functioning did not remain significant after correction for multiple testing.

This difference is important. A prenatal inflammatory profile may be related to later diagnostic risk without predicting a specific cognitive outcome.

### 5.4. Maternal Brain-Reactive Autoantibodies

Maternal brain-reactive autoantibodies provide another possible prenatal pathway. Maternal IgG normally crosses the placenta. If some antibodies recognize proteins expressed in the developing fetal brain, they may influence neurodevelopment.

Ramirez-Celis et al. [9] studied prospectively collected prenatal plasma from mothers of children later diagnosed with ASD, intellectual disability without ASD, or neither condition. One or more previously defined MAR-ASD antibody patterns were more common in the ASD group than in population controls. Some patterns also differed between ASD with and without intellectual disability.

Quantitative performance should be interpreted carefully. In the earlier assay-development study, several two-antigen MAR-ASD patterns were 100% specific for ASD in the study samples, with positive predictive values of 100% for several patterns and approximately 90–92% for two others; however, the patterns covered only about 18% of ASD cases in the training set and about 10% in the validation set [32]. In the independent prospective prenatal cohort, one or more of the nine predefined patterns were present in 10% of ASD cases, 4% of children with intellectual disability without ASD, and 1% of general-population controls (ASD vs. general population: OR 7.81, 95% CI 3.32–22.43) [9]. Thus, high pattern specificity coexists with low sensitivity/coverage. PPV and NPV also depend strongly on disease prevalence and cannot be transferred directly from enriched case–control samples to population screening. Antigen panels, assay platforms, positivity thresholds, batch calibration, and external population validation remain major barriers to clinical use.

Mazón-Cabrera et al. [33] identified additional maternal fetal-brain-reactive targets using a different discovery method. These results support the broader concept of maternal brain-reactive antibody subgroups, but antigen panels and laboratory methods are not yet standardized.

### 5.5. Maternal Autoantibodies and Phenotype

Direct links with neuropsychological phenotype remain limited. Angkustsiri et al. [34] found MAR-ASD patterns in a subgroup of mothers. Their children had greater autism symptom severity, but there were no significant differences in IQ or adaptive functioning.

This negative result is important. It shows that an immune-defined subgroup does not necessarily have lower global cognitive ability or poorer adaptive function. Different outcomes must be analyzed separately.

### 5.6. Experimental Evidence

Animal MAR-ASD models provide biological plausibility. Bruce et al. [35] reported changes in social behavior, vocalization, brain volume, and neurometabolites after gestational exposure to ASD-associated maternal autoantibodies. McLellan et al. [36] found that different maternal antibody patterns produced different early immune and transcriptomic changes in the offspring brain.

These studies show that maternal antibodies can influence development in experimental systems. They do not prove that the same mechanism explains the phenotype of individual autistic children.

### 5.7. Prenatal Immune Exposure in a Developmental Model

Overall, prenatal immune mechanisms are best understood as developmental risk modifiers. Human studies provide evidence of association, while animal studies provide stronger mechanistic evidence. The direct link from prenatal immune profile to specific long-term neuropsychological trajectory remains much less certain.

## 6. Postnatal Immune Dysregulation in Autism Spectrum Disorder

### 6.1. Peripheral Cytokines and Chemokines

Evidence of immune dysregulation continues after birth. Many studies have reported differences in circulating cytokines, chemokines, and other inflammatory markers, especially in children with ASD. Recent meta-analyses support group-level differences, but results remain heterogeneous.

Volk et al. [14] reported higher concentrations of several inflammatory mediators in autistic groups. Chen et al. [15] also found higher CRP, IL-6, IL-8, IL-1β, and BDNF at the group level. However, these measures are affected by many clinical and methodological factors and cannot currently be used as individual biomarkers.

Longitudinal evidence is rare. Kim et al. [24] showed that neonatal chemokines can precede later developmental differences. This supports the idea that early immune signals may relate to later development, but the direction and meaning of the relationship are not simple.

### 6.2. Innate Immune Cell Phenotypes

Monocytes are one possible link between peripheral immunity and neurodevelopment. Li et al. [16] found changes in monocyte populations and IL-6 production in children with ASD. Classical monocyte levels were negatively associated with selected developmental and adaptive quotients.

Because the study was cross-sectional, it cannot show whether immune changes cause developmental differences. Stress, medication, sleep, gastrointestinal problems, allergy, metabolic factors, or shared upstream mechanisms may also influence the findings.

### 6.3. Adaptive Immunity and T-Cell Regulation

Alterations in CD4+ and CD8+ T cells, TH1-, TH2-, TH17-related pathways, and regulatory T-cell function have been reported in ASD. Moreno et al. [37] reviewed this literature and highlighted altered T-cell activity and altered regulation of immune activation.

T cells also have roles outside classic immune defense. Experimental studies suggest that they can influence neurogenesis, microglia, neuronal maturation, and memory-related processes. However, direct human evidence linking T-cell profiles to specific cognitive trajectories in ASD is still limited and is mainly pediatric.

### 6.4. The Gut–Immune–Brain Interface

The gut is another possible source of neuroimmune modulation. Microbial metabolites can affect epithelial, immune, metabolic, and neural signaling. Morton et al. [38] identified coordinated relationships among microbiome, metabolites, cytokines, brain-related gene-expression patterns, and ASD phenotypic heterogeneity across several datasets.

These findings support a gut–immune–brain framework, but much of the evidence is cross-sectional. It is still unclear whether microbiome changes are a cause, consequence, or marker of broader biological differences.

Methodologically, microbiome studies also require analyses that respect the compositional nature of relative abundance data. Standard correlations on raw relative abundances can generate misleading associations because an increase in one taxon necessarily changes the relative proportion of others. Log-ratio or other validated compositional approaches, explicit handling of zeros, and sensitivity analyses for sequencing depth should therefore be considered [11]. Stronger causal inference also requires longitudinal sampling, temporally ordered exposure and outcome measures, household or sibling controls where possible, and intervention or natural experiment designs. These methods are more informative than cross-sectional taxon-by-taxon correlations.

Stronger causal inference will require longitudinal sampling, sibling or household controls, intervention studies, and approaches that test directionality rather than simple case–control differences. Multi-omics associations are useful for pathway discovery, but they should not be interpreted as proof that microbiome changes drive neuropsychological outcomes.

## 7. Neuroinflammation and Neural Circuit Development

### 7.1. From Microglial Activation to Microglial Heterogeneity

Early postmortem studies reported microglial and astroglial abnormalities and increased inflammatory markers in ASD. These studies helped establish the idea of central immune involvement. More recent evidence suggests that the term ‘microglial activation’ is too broad.

Microglia can have many functional states, and these states depend on region, developmental stage, sex, genetic background, metabolism, and immune exposure. Current evidence does not support one persistent microglial state across all autistic individuals.

### 7.2. Complement and Synaptic Remodeling

Complement proteins can mark synaptic elements for microglial recognition and removal during normal development. This gives complement an important role at the interface between immunity and neural circuit refinement.

Altered complement signaling could change excitatory–inhibitory balance and connectivity. However, direct human evidence for one specific complement-mediated pruning mechanism in ASD is limited. Most causal evidence comes from experimental systems.

Recent experimental work makes this point more specific. In SCN2A-deficient mice, complement C3 accumulated at post-synaptic sites and microglia showed excessive phagocytic pruning during selected developmental periods. A human cerebral organoid carrying an ASD-associated SCN2A mutation showed a similar increase in microglial elimination of post-synaptic elements [21]. In parallel, CRISPR-based studies in human microglia indicate that ASD risk genes such as ADNP can regulate endocytosis and synaptic pruning [20]. These studies provide an important bridge between genetic risk and immune-mediated circuit remodeling. However, they also show why the term “increased pruning” should not be generalized to all ASD. The direction of remodeling may depend on the gene, cell type, brain region, and developmental window.

The translational gap remains important: mouse and organoid models can demonstrate pathway-level causality under controlled conditions, but they cannot reproduce the full genetic, developmental, environmental, and clinical heterogeneity of human ASD. These results should therefore be used to support biological plausibility and mechanism, not direct clinical prediction.

### 7.3. Astrocytes and Inflammatory Signaling

Astrocytes regulate neurotransmitter and ion balance, support metabolism, influence synapse formation, and interact with microglia and neurons. Experimental studies suggest that astrocyte dysfunction may contribute to altered synaptic and inflammatory signaling in ASD-related models.

Shi et al. [39] linked inflammatory and pyroptosis-related markers in human ASD samples with an experimental S1P–NLRC4 astrocyte pathway. Suppression of this pathway improved selected cognitive and social outcomes in mice. The human causal chain, however, remains unproven.

### 7.4. Persistent Effects Do Not Require Persistent Neuroinflammation

Longitudinal MIA models show an important principle: an immune disturbance during development can have long-lasting brain or behavioral effects even if the original inflammatory process is no longer present.

Guerrin et al. [28] found increased brain glucose metabolism and adult recognition-memory deficits after prenatal poly(I:C), but did not find increased TSPO signal. Carbone et al. [29] found that behavioral changes and cytokine changes appeared at different ages after prenatal LPS exposure. These studies support a distinction between developmental immune programming and ongoing neuroinflammation.

TSPO-PET also has important technical and biological limitations. TSPO is an outer-mitochondrial-membrane protein and is not specific to activated microglia; signal can arise from astrocytes and other cellular compartments, and PET cannot distinguish functionally different glial states [18,40]. For several second-generation ligands, the rs6971 TSPO polymorphism strongly affects binding affinity and requires genotyping or appropriate stratification. Quantification is also influenced by tracer choice, kinetic modeling, reference-region assumptions, disease stage, and psychiatric comorbidity. These limitations may contribute to the inconsistent direction of ASD TSPO-PET findings. Future studies should combine genotype-aware PET with CSF or blood immune measures and, where possible, cell-resolved or spatial molecular approaches. New PET targets with greater cellular and functional specificity may also be needed.

### 7.5. Immunometabolism, Mitochondria, and Oxidative Stress

Immune cell function is closely linked to cellular metabolism. Microglia change their use of glucose, lipids, mitochondrial respiration, and lysosomal pathways according to developmental and inflammatory demands. In ASD-relevant models, altered lipid handling, mitochondrial function, and phagocytic-lysosomal load may therefore influence how microglia respond to synaptic and environmental signals [13,23]. This provides a mechanistic connection between immune signaling and metabolic abnormalities without assuming that all metabolic findings are inflammatory in origin.

Pediatric metabolomic studies also point to systemic pathways that may interact with immune regulation. In a 2024 case–control study of 59 children with ASD and 40 neurotypical children, 10 of 22 plasma one-carbon metabolites differed between groups. Children with ASD had a higher SAM/SAH ratio and a lower GSH/GSSG ratio, linking methylation and trans-sulfuration pathways with redox balance [41]. A separate untargeted plasma study in 28 children with ASD and 33 controls identified 116 differential metabolites and highlighted several lipid-related candidate markers, including fatty acid-derived metabolites [42]. These studies support metabolic heterogeneity, but they do not show that these metabolites cause immune dysregulation or specific cognitive phenotypes. Future work should measure metabolic, immune, and neuropsychological variables in the same cohort.

Mitochondrial dysfunction has also been reported in a subgroup of autistic individuals. A recent systematic review and meta-analysis identified abnormalities in mitochondrial biomarkers, mtDNA measures, respiration, lactate, carnitine, and related metabolic markers across heterogeneous studies [43]. Mitochondria are relevant to neuroimmune biology because they regulate ATP production, calcium handling, reactive oxygen species, and inflammatory signaling [44]. Oxidative stress can therefore act both as a consequence and as a modifier of immune and synaptic dysfunction. At present, however, mitochondrial or oxidative markers do not define a validated neuroimmune subtype of ASD, and associations with clinical severity are inconsistent across studies.

### 7.6. Blood–Brain Barrier and Neurovascular Mechanisms

The blood–brain barrier (BBB) and the neurovascular unit provide another possible interface between peripheral immunity and the developing brain. Endothelial cells, pericytes, astrocytic end-feet, and extracellular matrix components regulate transport, vascular signaling, and access of circulating inflammatory mediators to the central nervous system. Maternal inflammation and oxidative stress can also affect vascular and placental development, which makes neurovascular mechanisms relevant to prenatal models of ASD [45].

Evidence for BBB dysfunction in ASD is still much weaker than the evidence for peripheral immune differences or experimental MIA. Most detailed data come from animal models, postmortem observations, or broad neurovascular reviews. Direct human studies with validated measures of BBB permeability are scarce. For this reason, BBB disruption should be considered a plausible and testable mechanism rather than an established feature of ASD. Future studies should combine endothelial or permeability markers with immune phenotyping, imaging, and longitudinal neuropsychological measures in the same participants.

### 7.7. Glial Crosstalk, Engulfment Checkpoints, and Cellular Stress Pathways

Microglia and astrocytes should not be considered as independent inflammatory cell types. During development they exchange cytokines, metabolites, trophic signals, extracellular vesicles, and synapse-related cues. Astrocytes support synapse formation and neurotransmitter homeostasis, while microglia participate in synaptic surveillance and elimination. At the microglia-synapse interface, complement deposition is only one recognition system. Phosphatidylserine exposure can act as an ‘eat-me’ signal, whereas inhibitory checkpoints can limit engulfment. These parallel pathways help explain why altered synaptic remodeling in ASD may be excessive, insufficient, or mistargeted rather than uniformly increased [12,18].

Phagocytosis also depends on lysosomal and autophagic capacity. Increased synaptic cargo without adequate lysosomal processing can change microglial metabolism and inflammatory signaling, while impaired autophagy may affect both glial homeostasis and neuronal protein turnover. Recent ASD-relevant microglial work links genetic risk with endocytosis, lipid handling, lysosomal function, and inflammatory restraint [19,20,22,23]. These mechanisms provide a possible point of convergence between ASD genetics, immunometabolism, and circuit development. However, most causal evidence still comes from cellular or experimental systems, and the same pathways have not yet been validated as a common mechanism in human ASD.

## 8. Age-Related Neuropsychological Profiles in Autism Spectrum Disorder

### 8.1. Neuropsychological Heterogeneity Across Development

The neuropsychological profile of ASD changes across development and differs greatly between individuals. Relevant domains include language, attention, executive functions, memory, sensory processing, social cognition, emotion regulation, intellectual functioning, and adaptive behavior.

Cross-sectional age differences should not automatically be interpreted as longitudinal change. Cohorts may differ in diagnosis, intellectual ability, sex distribution, access to intervention, and participation. Longitudinal studies following the same individuals are still less common.

### 8.2. Childhood

Childhood is the period with the largest amount of direct immune–developmental evidence. Language, adaptive function, attention, sensory processing, and executive control show large variability. Li et al. [16] and Kim et al. [24] provide examples of direct associations between immune measures and developmental outcomes, but the evidence is not strong enough to define an immune-associated cognitive phenotype.

### 8.3. Adolescence

During adolescence, executive control, social cognition, emotional regulation, school demands, and autonomy become more important. Psychiatric comorbidity may also become more clinically relevant. Experimental MIA data suggest that developmental trajectories can change during this period and may differ by sex and environmental context [27].

### 8.4. Transition to Adulthood

Human data on the transition to adulthood are more limited. In the prospective ABIS cohort, young adults with ASD had more concentration difficulties than controls. The study also assessed health and psychosocial outcomes, but it did not show that concentration problems were caused by immune dysfunction [46].

### 8.5. Adult Compensation and Cognitive–Emotional Function

Adult ASD may include compensatory strategies such as camouflaging. Walsh et al. [47] studied adults aged 18–70 years and found sex-related brain connectivity patterns linked to camouflaging, cognitive control, and emotion recognition. However, the study did not measure inflammatory biomarkers. It is therefore useful for adult neuropsychology but not as direct evidence of an immune mechanism.

Psychiatric comorbidity, adaptive burden, and autistic burnout are also relevant in adulthood. These outcomes should be described as part of the adult phenotype without assuming an immune cause.

### 8.6. Older Adulthood and Aging

Research on aging in autistic adults has increased, but the available evidence is still much stronger for cognition, brain structure, and general health than for immune biology. A recent systematic review of 56 studies did not support a global pattern of accelerated cognitive or neural aging in autism. Most objective cognitive findings were similar to those in non-autistic adults, although subjective cognitive complaints and some white-matter findings suggested possible domain-specific vulnerabilities [48].

Longitudinal cognitive data also argue against a simple accelerated-aging model. Torenvliet et al. followed 128 autistic and 112 non-autistic adults aged 24–85 years over two to three assessments and found broadly parallel cognitive change across groups [49]. These findings are important for the present review because they show that age-related cognitive change in ASD cannot be assumed to reflect progressive neuroinflammation.

The main limitation is that these adult and aging studies rarely include detailed immune phenotyping. Therefore, the question is not whether cognitive aging occurs in ASD, but whether age-related immune remodeling modifies selected cognitive, adaptive, or psychiatric trajectories in identifiable subgroups. At present, direct longitudinal human evidence for this link is largely absent.

### 8.7. Autonomic Regulation as a Possible Adult Neuroimmune Interface

Autonomic regulation may provide one possible bridge between stress physiology, inflammation, and adult ASD. The vagus nerve contributes to parasympathetic control and is part of the cholinergic anti-inflammatory pathway. Heart-rate variability (HRV) is often used as a non-invasive marker of autonomic flexibility, although it is not a direct measure of inflammation.

In a study of 152 adults undergoing diagnostic assessment, including 56 adults with ASD, Villar de Araujo et al. found reduced parasympathetic and increased sympathetic activity in the ASD group compared with participants without a psychiatric diagnosis [50]. This finding supports autonomic dysregulation in at least some adults with ASD, but the study did not measure cytokines, immune cell profiles, or neuropsychological trajectories. HRV should therefore be considered a candidate systems-level bridge rather than evidence of an established adult neuroimmune mechanism.

Future adult studies could combine repeated HRV, inflammatory biomarkers, sleep and stress measures, medication exposure, and domain-specific cognitive or adaptive outcomes. Such designs would help test whether autonomic regulation modifies the relationship between systemic immune activity and neuropsychological function rather than simply correlating with ASD diagnosis.

## 9. Linking Immune Mechanisms to Neuropsychological Trajectories

### 9.1. Strong Biological Plausibility, Limited Direct Human Evidence

The immune literature and the neuropsychological literature are both large, but their direct overlap is small. Most immune studies focus on diagnosis or broad symptom severity. Most neuropsychological studies do not measure immune biology.

This creates an important methodological problem. A cytokine difference found in one study cannot be combined with executive dysfunction found in another study to conclude that the cytokine causes executive dysfunction. Both variables must be measured in the same individuals, ideally over time.

### 9.2. Direct Human Evidence

A small number of studies provide direct evidence. Li et al. [16] linked classical monocytes with selected adaptive and developmental quotients. Kim et al. [24] measured neonatal immune markers before later developmental characterization. Angkustsiri et al. [34] found that maternal MAR-ASD positivity was related to greater autism symptom severity but not to IQ or adaptive functioning. Ramirez-Celis et al. [9] found that some prenatal maternal autoantibody patterns differed between ASD with and without intellectual disability.

Together, these studies support selective associations rather than one general immune–cognitive relationship.

The small number of studies that directly measured immune markers and neuropsychological or developmental outcomes in the same human cohort are summarized in Table 2. The table also includes important negative findings, because a biomarker association with ASD diagnosis or symptom severity does not necessarily imply an association with IQ, executive function, or adaptive functioning.

### 9.3. Developmental Timing

Timing is likely to be critical. Prenatal immune exposure may influence neuronal proliferation, migration, glial maturation, and circuit formation. Postnatal immune changes occur in a nervous system that is already organized but still plastic.

Carbone et al. [29] and Guerrin et al. [28] show that immune and behavioral outcomes can appear at different times after one prenatal immune challenge. This makes it unlikely that one single inflammatory measurement can represent a person’s full developmental immune history.

### 9.4. Phenotypic Specificity

Autism symptom severity, IQ, executive function, language, social cognition, adaptive functioning, sensory processing, and psychiatric symptoms are different constructs. They should not be treated as interchangeable outcomes.

MAR-ASD studies are a good example. An association with autism symptom severity does not automatically mean lower IQ or worse adaptive function. Future studies should use clear and domain-specific neuropsychological outcomes.

### 9.5. Childhood Evidence Cannot Be Automatically Extended to Adulthood

The evidence base remains strongly age-asymmetric. Pediatric studies provide most of the direct same-cohort links between immune measures and developmental outcomes. In adulthood, the literature increasingly includes longitudinal cognition, neuroimaging, autonomic physiology, physical health, and quality of life, but these domains are usually studied without parallel immune measurements [47,48,49,50].

This distinction changes how the lifespan claim should be interpreted. The review can describe neuropsychological trajectories across the lifespan and immune mechanisms across developmental periods, but it cannot claim that specific immune profiles have already been shown to determine adult or aging trajectories. The strongest lifespan inference is therefore developmental and probabilistic: early immune perturbations can have persistent effects experimentally, while direct human evidence for ongoing immune-neuropsychological coupling in adulthood and aging remains a major gap.

### 9.6. Integrated Developmental Model

The current evidence is best explained by a probabilistic developmental model. Genetic susceptibility and prenatal exposure may influence placental signaling, maternal antibodies, fetal cytokines, and glial development. Postnatal immune cell function, cytokine networks, microbiome-derived metabolites, systemic comorbidities, and environmental exposures may then modify later development.

The final neuropsychological phenotype is also shaped by learning, intervention, sex-related biology, intellectual ability, psychiatric comorbidity, adaptive demands, and compensatory strategies. Immune mechanisms are therefore neither necessary nor sufficient to explain ASD. They may instead modify developmental outcomes in susceptible subgroups.

### 9.7. Sex-Related Differences and Neuroimmune Interpretation

Sex is an important source of heterogeneity in both ASD and neuroimmune biology. Experimental MIA and MAR-ASD studies show sex-dependent differences in behavior, brain structure, cytokine profiles, and developmental trajectories [25,28,29,35]. These findings support biological sex as a modifier of immune effects, but they should not be used to assume the same mechanisms in humans.

Human evidence is less direct. Adult neuroimaging studies suggest sex-related differences in connectivity and compensatory patterns, but detailed immune markers are usually not measured in the same participants [47]. Recent TSPO-PET work also suggests that sex may influence in vivo glial-associated signals, although samples remain small [18]. Future studies should therefore report sex-stratified results when adequately powered, test sex-by-biomarker interactions rather than only adjusting for sex, and distinguish biological sex from gender-related differences in diagnosis, masking, treatment, and environmental exposure.

### 9.8. Current Evidentiary Boundary

Three levels of inference can be separated. First, there is good evidence that immune dysregulation occurs in subsets of autistic individuals. Second, there is strong experimental evidence that immune disturbances can change neurodevelopment and produce persistent behavioral or cognitive effects. Third, there is much less human evidence that specific immune profiles determine specific neuropsychological trajectories across the lifespan.

This third level is the main unresolved question. At present, immune abnormalities should not be used to predict an individual’s cognitive outcome, and cognitive difficulties should not be assumed to indicate active inflammation.

**Figure 2:**
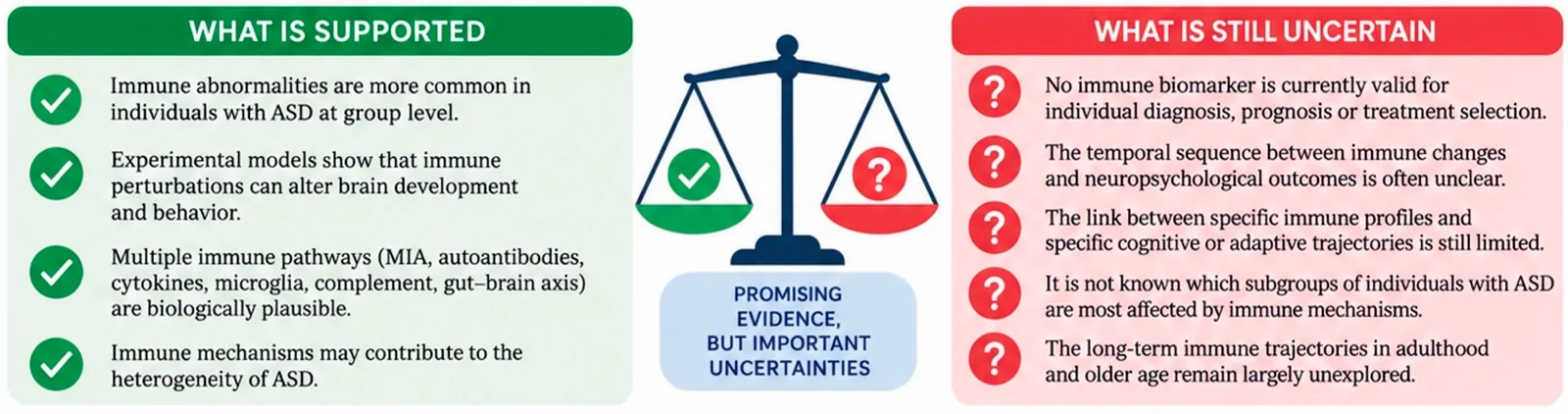
Current evidence linking immune mechanisms to neuropsychological trajectories in ASD. The strongest evidence concerns group-level immune differences and experimental developmental mechanisms, whereas direct human lifespan immune–cognition links remain limited.

The balance between the currently supported findings and the main remaining uncertainties is summarized in Figure 2.

Table 3 summarizes the main neuroimmune domains considered in this review, the type of supporting evidence, and the main limitations for interpretation.

To make the mechanistic synthesis more explicit, Table 4 separates human and experimental evidence according to developmental window, tissue or cell type, molecular pathway, associated phenotype, and level of causal support.

## 10. Clinical and Translational Implications

The evidence for immune involvement in ASD has possible clinical value, but biological relevance should not be confused with immediate clinical utility. Current findings support immune dysregulation as a possible modifier in some autistic individuals. They do not support routine use of inflammatory biomarkers for diagnosis, prognosis, or treatment selection.

Circulating cytokines, chemokines, immune cell profiles, and maternal autoantibody patterns are not validated individual-level ASD biomarkers. Group differences are reproducible in some studies, but there is large overlap between autistic and non-autistic people and many possible confounders [9,14,15,16].

Maternal autoantibody-related ASD is one of the more biologically defined candidate subgroups. Prospective studies support specific fetal brain-reactive antibody patterns, but antigen panels, laboratory methods, thresholds, and predictive values are not standardized for clinical screening [9,33,34,35,36].

The main translational value of neuroimmune research may therefore be the identification of biologically meaningful subgroups rather than the search for one universal autism biomarker. Future precision-medicine approaches may combine immune, genetic, metabolic, microbiome, neuroimaging, and neuropsychological data.

Therapeutic translation also requires caution. T-regulatory-cell strategies, cytokine-directed treatments, microglial or astrocytic targets, and microbiota-based interventions remain investigational. A clinically useful pathway should first identify a reproducible biological subgroup, show that the biological feature predicts a meaningful outcome, and then show that modifying the pathway improves that outcome.

## 11. Limitations of Current Evidence

Several limitations affect the neuroimmune literature in ASD. First, study populations are very heterogeneous. Age, sex, intellectual functioning, language, psychiatric comorbidity, epilepsy, gastrointestinal and allergic disorders, medication, body mass index, sleep, infection, and socioeconomic factors can all influence immune measures.

Second, immune measurements are also heterogeneous. Studies use serum, plasma, cerebrospinal fluid, neonatal blood spots, postmortem tissue, maternal plasma, and stimulated cell assays. These biological compartments are not equivalent. A peripheral cytokine level does not directly measure central nervous system inflammation.

Third, most clinical studies are cross-sectional. It is often impossible to know whether an immune difference comes before, contributes to, accompanies, or follows the neurodevelopmental phenotype.

Fourth, neuropsychological outcomes are defined in different ways. Autism severity, IQ, executive function, language, adaptive function, sensory processing, and psychiatric symptoms are sometimes combined even though they represent different constructs.

Fifth, direct immune–neuropsychological studies are rare. This problem is particularly clear in adulthood. Adult ASD studies describe concentration difficulties, camouflaging, cognitive control, emotion recognition, adaptive burden, and psychiatric comorbidity, but they rarely include detailed immune biomarkers.

Sixth, translation from experimental models is difficult. MIA and maternal autoantibody models provide important causal evidence, but they use controlled timing, dose, genetics, and environment. Human ASD is much more heterogeneous.

Accordingly, throughout this review, animal and in vitro findings are interpreted as experimental evidence unless a corresponding human association is available. Experimental rescue or pathway manipulation is not considered proof of therapeutic relevance in unselected individuals with ASD.

Finally, publication bias and selective reporting are possible, especially in small biomarker studies that measure many analytes. Independent replication and better harmonization are needed.

## 12. Future Research Directions

Future studies should move beyond average immune differences between autistic and non-autistic groups. The key question is whether immune variation explains meaningful differences among autistic individuals.

The highest priority is prospective longitudinal phenotyping. Immune and neuropsychological measures should be repeated in the same individuals from prenatal or neonatal periods through childhood, adolescence, and adulthood whenever possible.

Neuropsychological assessment should also be more detailed. Studies should separate intellectual ability, language, attention, memory, executive control, social cognition, sensory processing, emotion regulation, and adaptive functioning.

Immune assessment should move beyond single cytokines. Network approaches that combine cytokines, immune cell phenotypes, transcriptomics, proteomics, metabolomics, autoantibodies, microbiome-derived metabolites, and genetic susceptibility may provide more useful biological profiles.

Developmental timing should be treated as a main variable. Prenatal immune exposure, neonatal immune state, childhood inflammation, adolescent immune remodeling, and adult immune function are different biological contexts.

Sex and gender also need more attention. Many animal studies remain male-only, while many human studies are too small for reliable sex-stratified analysis.

Adult and aging research is a major unmet need. A useful next step would be a prospective cohort spanning early adulthood to older age with repeated assessments every 2–3 years. The same participants should undergo domain-specific cognitive testing, standardized adaptive-function measures, psychiatric assessment, HRV or other autonomic measures, blood cytokine and immune cell profiling, metabolic measures, medication and comorbidity recording, and, in a subset, neuroimaging. This design would allow for within-person analysis of whether changes in immune or autonomic state precede, follow, or occur independently from changes in cognition and adaptive functioning.

Finally, candidate biological subgroups should be validated prospectively before they are used for treatment selection. Better biospecimen standards, assay harmonization, reporting of medication and comorbidities, preregistration, correction for multiple testing, and independent replication should become routine.

## 13. Conclusions

ASD is a heterogeneous neurodevelopmental condition in which genetic, environmental, neural, and immune factors can interact across development. Current evidence shows that immune-related mechanisms are relevant in at least a subgroup of autistic individuals.

Maternal immune activation, maternal brain-reactive autoantibodies, peripheral cytokine and immune cell changes, microglial and astrocytic processes, complement-mediated synaptic remodeling, and gut–immune–brain pathways provide possible links between immune biology and neurodevelopment.

However, the strength of evidence is not the same at all levels. Group-level immune differences are relatively well supported, and experimental models show that immune disturbances can modify brain development and later behavior. Direct human evidence linking specific immune profiles with specific neuropsychological trajectories is much weaker.

The lifespan framework should therefore be interpreted as a developmental model with unequal evidence across ages: mechanistic and direct clinical evidence is strongest in prenatal and pediatric periods, while adult and aging neuroimmune trajectories remain insufficiently studied.

This difference is especially clear across the lifespan. Most direct immune–phenotype studies are pediatric, while adult neuropsychological studies rarely include detailed immune measurements. Experimental MIA studies show that prenatal immune disturbances can have delayed effects, but persistent behavioral changes do not necessarily mean persistent neuroinflammation.

Current evidence therefore supports a probabilistic rather than a deterministic model. Immune abnormalities are unlikely to be a universal cause of ASD. They may instead interact with genetic susceptibility, developmental timing, sex, metabolism, microbiome, environmental exposure, and neural plasticity to influence outcomes in some biological subgroups.

The main future challenge is to connect molecular immunology with careful longitudinal neuropsychological assessment. This approach may support better biological stratification, biomarker development, prognosis, and eventually more personalized interventions.

## Figures and Tables

**Table 1:** Practical recommendations for immune biomarker studies in ASD.

Domain	Recommended Practice
Sampling and timing	Record fasting status, time of day, acute stress, sleep, and recent infection. Use a pre-specified exclusion or deferral window for acute febrile/infectious illness rather than mixing acutely ill and clinically stable participants.
Serum vs. plasma	Do not treat serum and plasma values as interchangeable. Report anticoagulant type, clotting time where relevant, processing delay, centrifugation conditions, storage temperature, and number of freeze–thaw cycles.
Assay platform	State whether measurements are single-plex or multiplex, the platform and kit, lower limit of detection, values below detection, duplicate variability, batch structure, and quality-control procedures. Randomize diagnostic groups across plates/batches when possible.
Core confounders	Record age, sex, BMI, medication, allergy/atopy, gastrointestinal disease, epilepsy, sleep, recent infection, and major psychiatric or medical comorbidity. Pre-specify which factors are exclusion criteria, stratification variables, or covariates.
Statistical analysis	Pre-specify primary biomarkers/outcomes, report effect sizes and confidence intervals, correct for multiple testing, and report negative results. Avoid selecting cut-offs in the same small sample used to estimate diagnostic performance.
Microbiome data	Account for compositionality, sequencing depth, and zero inflation. Use methods appropriate for compositional data, and perform sensitivity analyses for diet, antibiotics, household/sibling structure, and stool-processing variables [11].
Neuropsychological outcomes	Use domain-specific standardized measures. Keep autism symptom severity, IQ, executive function, language, social cognition, and adaptive functioning separate. When adaptive functioning is an outcome, report standardized instruments such as the Vineland scales where available.
Longitudinal design	Use repeated immune and neuropsychological measures in the same participants. This allows for testing of temporal direction and within-person change, which cross-sectional case–control designs cannot establish.

**Table 2:** Key human studies directly linking immune markers or prenatal immune exposures to neuropsychological or developmental outcomes in ASD.

Study	Design/Sample	Immune Measure/Biospecimen	Neuropsychological or Developmental Outcome	Main Association/Direction	Key Limitation
Kim et al. [24]	Longitudinal; 398 children	Neonatal blood spots; chemokines including CCL23/MPIF-1 and CCL27/CTACK	Mullen developmental domains and Vineland adaptive domains	Neonatal chemokine patterns preceded later diagnosis; higher MPIF-1 was associated with better scores in selected domains	Exploratory markers/multiple testing; peripheral neonatal sample; replication needed
Li et al. [16]	Cross-sectional pediatric immune phenotyping; ASD *n* = 92, TD *n* = 96	Peripheral monocyte subsets; IL-6/IL-10	Adaptive behavior DQ, fine-motor DQ, personal–social DQ	Higher classical monocyte levels were associated with lower scores in selected adaptive/developmental domains	Cross-sectional; residual confounding; replication needed
Angkustsiri et al. [34]	Pilot multisite human study; 68 mothers/children	Maternal plasma; MAR-ASD autoantibody patterns	ADOS/SCQ severity, IQ, adaptive functioning	MAR-ASD positivity was associated with greater autism symptom severity, but not with IQ or adaptive functioning	Small sample; maternal plasma collected years after pregnancy
Ramirez-Celis et al. [9]	Prospective prenatal biomarker study; ASD *n* = 540, ID *n* = 184, controls *n* = 420	Prenatal maternal plasma; predefined MAR-ASD patterns	ASD diagnosis and ASD with/without intellectual disability	Patterns in 10% ASD vs. 1% controls; some patterns differed by ID status	Low sensitivity/coverage; rare patterns; no domain-specific cognitive battery
Wang et al. [31]	Prospective birth cohort; 555 mother-child pairs	Maternal plasma at gestational week 24; 92 inflammatory proteins	NDD diagnosis and executive function at age 10	Inflammatory PC1 associated with NDD/ASD risk; no EF association remained after FDR correction	Diagnostic association did not translate to robust EF association

**Table 3:** Summary of neuroimmune evidence in autism spectrum disorder.

Domain	Representative Evidence	Main Message	Main Limitation
Peripheral cytokines/chemokines	Meta-analyses and biomarker studies	Group-level immune differences are present in ASD.	Not validated as individual biomarkers; phenotype links are inconsistent.
Monocyte phenotypes	Pediatric observational studies	Monocyte abnormalities may relate to adaptive and developmental functioning.	Evidence is mainly cross-sectional.
T-cell regulation	Integrative immune studies	T-cell dysregulation is biologically plausible in ASD.	Direct neuropsychological linkage is limited.
Gut–immune–brain axis	Multi-omics and mechanistic studies	Microbial and metabolic signals may modulate immune and brain pathways.	Causality remains unclear.
Maternal immune activation	Human risk studies and preclinical models	Prenatal immune exposure may modify neurodevelopment.	Human causal mechanisms remain uncertain.
Maternal autoantibodies (MAR-ASD)	Prospective prenatal and mechanistic studies	A biologically defined subgroup is supported.	Not standardized or validated for clinical screening.
Adult and aging evidence	Adult cohort, immune, and neuroimaging literature	Adult neuropsychology and immune findings are rarely measured together.	Major evidence gap, especially for longitudinal and older-adult data.

**Table 4:** Mechanistic synthesis of neuroimmune evidence in ASD across developmental windows.

Developmental Window	Evidence/Model	Tissue or Cell Type	Molecular Pathway	Associated Phenotype/Outcome	Causal Support
Prenatal	Human prospective maternal biomarker studies [9,31]	Maternal plasma/placenta-related exposure	MAR-ASD autoantibodies; multivariate inflammatory proteins	ASD risk; ASD with/without ID; no robust EF association for maternal inflammatory PC1	Temporal human association; mechanism not proven
Prenatal	MIA animal models: poly(I:C), LPS [25,26,27,28,29]	Maternal immune system, placenta, fetal brain, microglia	IL-6/IL-17A in selected models; TLR-related signaling; fetal cytokines; glial programming	Social, cognitive, metabolic, and sex-dependent developmental outcomes	Experimental causality; strong model dependence
Prenatal/early development	MAR-ASD experimental models [35,36]	Maternal IgG; fetal brain	Pattern-specific autoantibody exposure; cytokines; growth factors; transcriptomic programs	Vocalization, social behavior, brain volume, neurometabolites, early gene-expression changes	Experimental causality; human translation indirect
Neonatal/childhood	Human neonatal biomarker study [24]	Neonatal blood spots	CCL23/MPIF-1, CCL27/CTACK and related chemokines	Later ASD/DD classification and selected Mullen/Vineland developmental domains	Temporal human association; not clinically predictive
Childhood	Human immune phenotyping [16]	Peripheral monocytes	Monocyte subsets; IL-6/IL-10 responses	Adaptive behavior, fine-motor and personal–social developmental quotients	Direct same-cohort association; cross-sectional
Developmental	ASD risk gene models in human microglia/organoids and mice [19,20,21,22,23]	Microglia; cerebral organoids; synapses	ADNP; SCN2A; MEF2C; complement C3; endocytosis; lysosome/lipid handling	Synaptic engulfment, microglial inflammatory state, circuit remodeling	Strong experimental/cellular causality; limited human in vivo validation
Postnatal	Human + experimental astrocyte study [39]	Peripheral blood markers; astrocytes; hippocampal circuits	S1P-NLRC4 inflammasome; pyroptosis; IL-1β/IL-18	Cognitive and social phenotypes in mice; human biomarker association	Causal rescue preclinical; human chain associative
Childhood to adulthood	Human cytokine meta-analyses [14,15]	Peripheral blood	IL-1β, IL-6, IL-8, TNF-α, IFN-γ and related markers	Group-level ASD differences; weak and inconsistent within-ASD phenotype linkage	Strong group association; low individual prognostic specificity
Postnatal/lifespan	Human multi-omics gut–brain study [38]	Gut microbiome, metabolites, cytokines, transcriptomic datasets	Microbial-metabolic-immune networks	ASD phenotypic heterogeneity; longitudinal microbiome changes in a subset	Systems-level association; causal direction unresolved
Adulthood/aging	Adult ASD cohort, autonomic, cognitive aging, and neuroimaging literature [37,46,47,48,49,50]	Peripheral/clinical data; brain connectivity	T-cell biology discussed indirectly; autonomic dysregulation/HRV; no integrated immune-cognition biomarker framework	Concentration difficulties, compensation/camouflaging, cognitive aging, autonomic differences	Major evidence gap; direct immune-neuropsychological causality not established

Note: ‘Causal support’ refers to the level of inference supported by the cited evidence and does not represent a formal GRADE rating. ID, intellectual disability; EF, executive function; MIA, maternal immune activation.

## Data Availability

No new data were created or analyzed in this study. Data sharing is not applicable to this article.

## Supplementary Materials

The following supporting information can be downloaded at: https://www.mdpi.com/article/10.3390/ijms27188407/s1.

## Glossary and Abbreviations

Glossary of key neuroimmune terms:

**Term**	**Working Definition Used in This Review**
Immune dysregulation	Altered immune cell function, signaling, regulation, or response; it does not necessarily imply systemic inflammation.
Systemic inflammation	Inflammatory activity measured outside the central nervous system, for example in blood or peripheral tissues.
Neuroinflammation	Inflammatory or immune-related processes within the central nervous system. Peripheral cytokine changes alone are not sufficient to demonstrate neuroinflammation.
Maternal immune activation (MIA)	Maternal inflammatory response during pregnancy caused by infectious or non-infectious immune stimuli; experimental MIA paradigms vary by stimulus, dose, timing, and model.
MAR-ASD	Maternal autoantibody-related ASD; a proposed biologically defined subgroup associated with specific maternal fetal brain-reactive IgG patterns.
Direct clinical evidence	Human evidence in which an immune exposure/measure and a neuropsychological or developmental outcome are linked within the same cohort.
Experimental evidence	Animal, cellular, organoid, or other model evidence in which an immune pathway is manipulated or mechanistically tested.

The following abbreviations are used in this manuscript:

ABIS	All Babies in Southeast Sweden
ASD	Autism spectrum disorder
BDNF	Brain-derived neurotrophic factor
CRP	C-reactive protein
IFN-γ	Interferon gamma
IgG	Immunoglobulin G
IL	Interleukin
LPS	Lipopolysaccharide
MAR-ASD	Maternal autoantibody-related autism spectrum disorder
MIA	Maternal immune activation
MIF	Macrophage migration inhibitory factor
NLRC4	NLR family CARD domain containing 4
S1P	Sphingosine-1-phosphate
TNF-α	Tumor necrosis factor alpha
TSPO	18 kDa translocator protein
