# Supplementary material for: Immune and Autoimmune Mechanisms in Autism Spectrum Disorder: Linking Molecular Pathways to Neuropsychological Trajectories Across the Lifespan
Source: Int J Mol Sci. 2026 Sep 21;27(18):8407. doi: 10.3390/ijms27188407 (PMC13607567; doi:10.3390/ijms27188407)
Supplement: Supplementary file 1 [file ijms-27-08407-s001.zip › ijms-4541218-supplementary.pdf]

Supplementary Material

Supplementary Table S1. Reconstructed PubMed search strategies

All searches were restricted in PubMed using the “last 5 years” filter. The original PubMed History log was not retained. The strings below were reconstructed from the documented thematic modules, saved PubMed exports, and screening files; they are reproducible reconstructions rather than an archived verbatim query log.

Module	Reconstructed PubMed string	Filter	Purpose
Core ASD–neuroimmune	("autism spectrum disorder"[Title/Abstract] OR autism[Title/Abstract] OR ASD[Title/Abstract]) AND (immune[Title/Abstract] OR immun*[Title/Abstract] OR inflammation[Title/Abstract] OR neuroinflammation[Title/Abstract] OR autoimmune*[Title/Abstract])	Last 5 years	Broad immune/autoimmune ASD literature
Maternal immune activation	("autism spectrum disorder"[Title/Abstract] OR autism[Title/Abstract] OR ASD[Title/Abstract]) AND ("maternal immune activation"[Title/Abstract] OR "maternal inflammation"[Title/Abstract] OR prenatal inflammation[Title/Abstract] OR MIA[Title/Abstract])	Last 5 years	Prenatal inflammatory exposure
Maternal autoantibodies / MAR-ASD	("autism spectrum disorder"[Title/Abstract] OR autism[Title/Abstract] OR ASD[Title/Abstract]) AND (maternal autoantibod*[Title/Abstract] OR "brain-reactive autoantibodies"[Title/Abstract] OR "fetal brain"[Title/Abstract] OR MAR-ASD[Title/Abstract])	Last 5 years	Maternal autoantibody patterns/phenotype
Peripheral cytokines / immune cells	("autism spectrum disorder"[Title/Abstract] OR autism[Title/Abstract] OR ASD[Title/Abstract]) AND (cytokine*[Title/Abstract] OR chemokine*[Title/Abstract] OR monocyte*[Title/Abstract] OR "T cell"[Title/Abstract] OR "T-cell"[Title/Abstract] OR immune-cell*[Title/Abstract])	Last 5 years	Peripheral inflammatory/cellular profiles
Microglia / astrocytes / neuroinflammation	("autism spectrum disorder"[Title/Abstract] OR autism[Title/Abstract] OR ASD[Title/Abstract]) AND (microglia*[Title/Abstract] OR astrocyte*[Title/Abstract] OR neuroinflammation[Title/Abstract] OR glial[Title/Abstract])	Last 5 years	Central glial mechanisms
Complement / synaptic remodeling	("autism spectrum disorder"[Title/Abstract] OR autism[Title/Abstract] OR ASD[Title/Abstract]) AND (complement[Title/Abstract] OR C1q[Title/Abstract] OR C3[Title/Abstract]) AND (synap*[Title/Abstract] OR pruning[Title/Abstract] OR remodeling[Title/Abstract])	Last 5 years	Complement and synaptic refinement
Autoimmune comorbidity	("autism spectrum disorder"[Title/Abstract] OR autism[Title/Abstract] OR ASD[Title/Abstract]) AND (autoimmune*[Title/Abstract] OR autoimmunity[Title/Abstract] OR "rheumatoid arthritis"[Title/Abstract] OR "immune-mediated"[Title/Abstract])	Last 5 years	Autoimmune context
Direct immune–neuropsych links	("autism spectrum disorder"[Title/Abstract] OR autism[Title/Abstract] OR ASD[Title/Abstract]) AND (immune[Title/Abstract] OR cytokine*[Title/Abstract] OR chemokine*[Title/Abstract] OR monocyte*[Title/Abstract] OR autoantibod*[Title/Abstract]) AND (cognit*[Title/Abstract] OR neuropsycholog*[Title/Abstract] OR executive[Title/Abstract] OR language[Title/Abstract] OR adaptive[Title/Abstract] OR "social cognition"[Title/Abstract])	Last 5 years	Same-cohort immune and neuropsych outcomes
Adult / aging / lifespan	("autism spectrum disorder"[Title/Abstract] OR autism[Title/Abstract] OR ASD[Title/Abstract]) AND (adult*[Title/Abstract] OR aging[Title/Abstract] OR ageing[Title/Abstract] OR lifespan[Title/Abstract] OR longitudinal[Title/Abstract]) AND (immune[Title/Abstract] OR inflammation[Title/Abstract] OR cytokine*[Title/Abstract] OR microglia*[Title/Abstract])	Last 5 years	Adult and longitudinal evidence

Supplementary Table S2. Design-adapted methodological appraisal of the 32 full-text papers

Overall confidence is a narrative weighting (higher, moderate, lower), not a GRADE rating. Appraisal domains were adapted to each study design.

Study	Design	Main methodological issues	Overall confidence
E01 Bouelkhair 2026	Systematic review	Heterogeneous models/assays; limited ASD-specific human mechanism	Moderate
E02 Al-Salihy 2026	Scoping review	No formal RoB; conceptual subtype framework	Moderate
E03 Jäntti 2026	Mechanistic review	Model/region/sex/stage heterogeneity	Moderate
E04 Moreno & Ashwood 2026	Viewpoint	Not systematic; preliminary therapeutic evidence	Lower
E05 Russell & Perreault 2026	Narrative MIA review	Predominantly preclinical; model variability	Moderate
E06 Yong 2025	Integrative MIA review	Mostly preclinical; mixed NDDs/species/timing	Moderate
E07 Schiano-Visconte 2026	Focused review	Human causality not established	Moderate
E08 Kurmashev 2026	Transcriptomic synthesis	Postmortem/platform/cell-composition effects	Moderate
E09 Lian 2026	Microglia review	Human data mainly associative	Moderate
E10 Zwierko 2026	Structured narrative review	Few same-cohort immune+fMRI studies	Lower–moderate
E11 Leisman 2026	Integrative review	Narrative cascade; mixed evidence levels	Moderate
E12 Cui 2026	Astrocyte review	Mixed idiopathic/syndromic models; therapy preclinical	Moderate
E13 Chen 2026	Systematic review/meta-analysis	Between-study and assay heterogeneity	Higher
E14 Varia 2026	Broad narrative review	Non-systematic, very broad scope	Lower
E15 Zhu 2025	National cohorts + meta-analysis	Residual confounding; medication/paternal RA limits	Higher
E16 Volk 2026	Systematic review/meta-analysis	Underlying study RoB; assay/sample heterogeneity	Higher
E17 Ahrens 2024	Prospective birth cohort	Subset biospecimens; residual confounding; no formal neuropsych battery	Higher
E18 Schaer 2025	Longitudinal MIA mouse study	Model-specific; sex-dependent; limited behavioral sampling	Moderate
E19 Morton 2023	Multi-omic human study	Mostly cross-sectional; platform/household effects	Moderate
E20 Li 2025	Cross-sectional pediatric immune study	Cross-sectional; residual confounding; replication needed	Moderate
E21 Kim 2022	Neonatal longitudinal biomarker study	Exploratory markers/multiple testing; peripheral sample	Higher
E22 Shi 2026	Human biomarker + animal/in vitro	Human evidence associative; rescue preclinical	Moderate
E23 Angkustsiri 2022	Pilot MAR-ASD human study	Small sample; post-pregnancy plasma; no non-ASD maternal controls	Lower–moderate
E24 Mazón-Cabrera 2023	Human autoantibody study	Post-pregnancy samples; discovery-platform limits	Moderate
E25 Ramirez-Celis 2022	Prospective prenatal biomarker study	Rare patterns; imprecise subgroup estimates; no clinical validation	Higher
E26 Bruce 2023	Longitudinal MAR-ASD rat model	Preclinical; limited sample size; target effects incomplete	Moderate
E27 McLellan 2025	Pattern-specific MAR-ASD rat study	Limited power; plate variation; some uncorrected p values	Moderate
E28 Moreno 2025	Integrative T-cell review	Direct evidence mostly pediatric; adult implications inferential	Moderate
E29 Walsh 2023	Adult multimodal neuroimaging	Small sample; broad age range; no immune biomarker	Moderate for neuropsychology; lower for immune linkage
E30 Lebeña 2023	Prospective cohort age 17–19	Self-report/attrition; no immune biomarker–cognition analysis	Higher for adult outcomes; lower for immune linkage
E31 Carbone 2023	Longitudinal prenatal-LPS rat study	Male-only; small cytokine sample; no direct immune–behavior causality	Moderate
E32 Guerrin 2022	Longitudinal MIA PET/behavior rat study	Small samples; no multiple-test correction; male-only	Moderate

Supplementary Figure S1. PRISMA-style flow diagram of the literature search and selection process

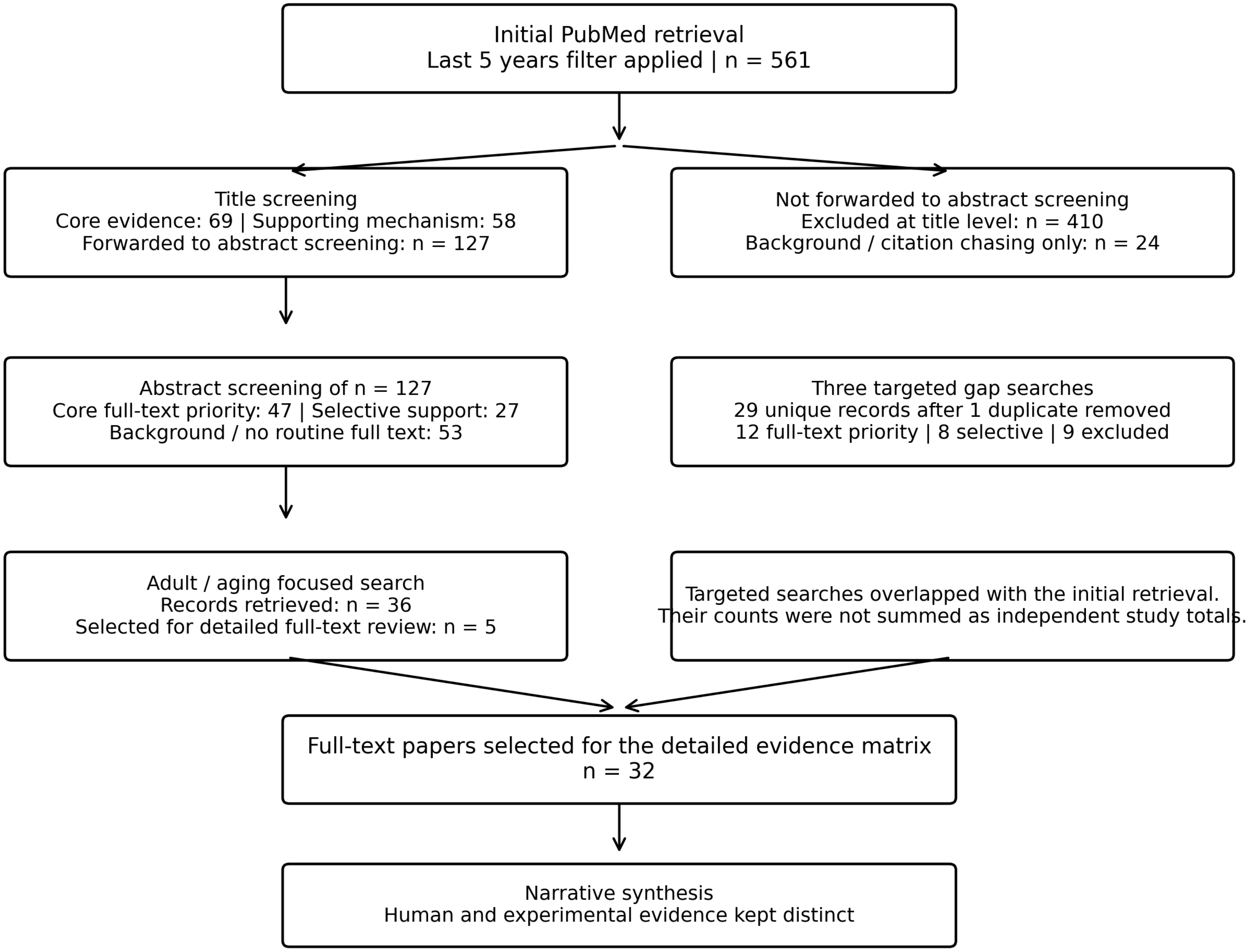

Legend: All PubMed searches used the "last 5 years" filter. The diagram is provided to improve transparency of the search and study-selection process in this narrative review. The targeted searches overlapped with the initial retrieval, so their counts should not be summed as independent study totals. Screening was not performed as a formal dual-independent systematic-review process.
